# Herpes simplex virus and varicella zoster virus, the house guests who never leave

**DOI:** 10.1186/2042-4280-3-5

**Published:** 2012-06-12

**Authors:** Paul R Kinchington, Anthony J St Leger, Jean-Marc G Guedon, Robert L Hendricks

**Affiliations:** 1 Department of Ophthalmology, University of Pittsburgh School of Medicine, Pittsburgh, PA, USA; 2 Department of Molecular Genetics & Biochemistry, University of Pittsburgh, Pittsburgh, PA, USA; 3 Department of Immunology, University of Pittsburgh School of Medicine, Pittsburgh, PA, USA

**Keywords:** Alphaherpesvirus, Herpes Simplex Virus, HSV, Varicella Zoster Virus, VZV, Immunity, Pathogenesis

## Abstract

Human alphaherpesviruses including herpes simplex viruses (HSV-1, HSV-2) and varicella zoster virus (VZV) establish persistent latent infection in sensory neurons for the life of the host. All three viruses have the potential to reactivate causing recurrent disease. Regardless of the homology between the different virus strains, the three viruses are characterized by varying pathologies. This review will highlight the differences in infection pattern, immune response, and pathogenesis associated with HSV-1 and VZV.

## Introduction

Herpes simplex viruses (HSV-1, HSV-2) and varicella zoster virus (VZV) are related human alphaherpesviruses that cause common, self-resolving diseases of the skin or mucosa, and concurrently establish a persistent latent infection of neuronal nuclei in the sensory ganglia innervating the peripheral site of infection. All three viruses may subsequently reactivate to cause recurrent disease in the face of existing immunity. At the molecular level, the three viruses share most genes and encode similar functions. Given these observations, it is perhaps surprising that HSV and VZV adopt very different modes of pathogenesis in the human host. Each virus has evolved a unique infection pattern and has developed specific abilities to counteract host innate and adaptive immunity. This review will highlight some remarkable differences in pathogenesis of these viruses, focusing on their interaction with host immunity. We will build on recent developments and previous reviews comparing these two herpesviruses [1,2]. While recognizing that significant differences exist between the two serotypes of HSV, this review will largely focus on a comparison of HSV-1 and VZV (Table 1).

**Table 1:** Comparison of HSV and VZV with illustrations of differences and similarities

**Sections**		**HSV-1**	**VZV**
**Viral**	Genes	~80: 4 diploid (ICP4, 0, 34.5, LAT)	68: 3 diploid (ORFs 62, 63, 64)
**Genome**	Size	~152kbp	~125kbp
	G + C content	−67%	−47%
	Repeats	-Large repeats for both UL and US	-Large on US only; 88.5 bp on UL
	Isomers	4	Mostly 2 with UL region fixed
	miRNA	From LAT region- role not yet clear	No known miRNAs
**Viral Proteins**	regulation	Regulated Cascade –defined as	Likely similar, but difficult to define experimentally
	Immediate Early differences		
		α, β1, β2, γ1, γ2-six genes (ICP0, ICP4, ICP27, ICP22, ICP1.5, ICP47.	-Three genes reported to date
		-All have TAATGARAT motif in IE promoters	-ORF/IE62(ICP4 Eeq) ORF/IE4(ICP27 eq) and ORF/IE63 (ICP22 eq)
			-No ortholog of ICP47.
			-Only IE62 has TAATGARAT in promoter
	Short Region differences	-gD, an essential protein involved in receptor & entry	-No gD, is essential-
		-gE not required in culture	-gE is key receptor binding protein
			-Missing several HSV equivalents
	Tegument differences	-UL48 (VP16) required in culture:	-ORF10(VP16 Eq) not required in culture:
		-UL49 not required	-ORF9 (UL49 eq) required
**Primary Infection**	Route of Infection	Spread through direct contact.	Spread via aerosol and inhalation.
	Location of 1^o^ infection	-Epithelia in mucosa, cornea or in epidermal layers of the skin	-Epithelial and immune cells in respiratory lymphoid tissues, tonsils
		-Usually no viremia	-Cell associated viremia
			-Secondary infection at the sub-dermis
	Spread to neurons	-Usually local only	-Systemic across entire neuraxis
		-Accesses neuronal axon termini in skin	-Same as HSV; may also access neurons during viremia thrugh immune cells
	Innate	TLR-2,3,9 respond to infection	Thought to be the same, but not known
		IFN regulates infection	IFN regulates infection
		NO helps retard viral replication	Role of NO not known
		ICP0 degrades PML and ND10 proteins	Susceptible to PML caging.
			ORF61 modifies ND10, does not degrade PML
**Innate and adaptive immunity**	Adaptive T cell response	CD4 and CD8 encounter antigen on DCs and respond to infection	-T cells infected by VZV leading to viral spread.
			-CD4 and CD8 T cells are VZV specific
	DC	Can infect and reduce presentation to T cells by DCs	-Can infect and reduce presentation to T cells by DCs
	Humoral Response	Elicit antibodies against broad viral antigens. IgA, IgG and IgM	-Elicit antibodies against broad viral antigens. IgA, IgG and IgM.
			-Antibodies are used in high risk patients to treat VZV
			-Antibody has less role on control of infection/ latency and reactivation
	Immune Evasion	ICP47 blocks TAP function.	-Does not block TAP function.
			-Still blocks MHCI and II expression.
			-Blocks MHCI by ORF66 kinase
		Inhibit IFN responses thru VHS, ICP0, and γ34.5	-Inhibit IFN responses by IE63, IE62
			-ORF61 blocks NFkB signaling
		gC blocks complement deposition	No equivalent activity for gC
		Fc binding ability of gE	VZV gE and gI complex to bind Fc
		ICP22, Us5, Us3 and LAT inhibit apoptosis by NK and CD8+ cell mediated lysis	ORF63 blocks apoptosis
**Models and Neuronal Latency**	Animal modeling	-Most animal models replicate virus	-Guinea pig only small natural animal model that replicates virus
		-Most show similar disease to humans	-No natural model of varicella
			-No model of reactivated disease
	Location of latency	Sensory ganglia, especially trigeminal ganglia	-Most sensory and autonomic ganglia
			-Distributed across entire neuraxis
	Load	Generally higher genome load than VZV	About one magnitude lower genome load
**Maintenance latency**		-Endless Circular episome.	-Endless circular episome.
		-Heterochromatinated state	-Assumed to be Heterochromatinated state
	Latent Gene Expression	-Abundant transcripts from LAT region	-RNAs for ORFs 4,21,61,10,29,62,63, and 66. --Reported protein expression is controversial
		-LATs processed into miRNAs	-ORF63 most often reported as expressed
		-LATs block apoptosis	
		-Rare protein expression without virus	
	Immune Component	-Drives ganglionic CD8+ immune infiltrate	-No Immune infiltrate yet reported
		-CD8 may control reactivation events	-Cellular immunity maintains latency
**Reactivation and disease**	Occurrence	-May Reactivate frequently	-Reactivated disease usually never or once
		-Incidence drops with age	-Incidence rises with age and declining cellular immunity.
		-Disease similar to primary infection	-Occurs anywhere on body
		-At same site as 1^o^ infection	-Disease clinically different from 1^o^ Infection
	Ganglionic Spread	Involves 1 or few neurons	-Usually intraganglionic spread
			-Large lesions covering a dermatome.
	Causes of reactivation	Multiple environmental and physiological factors	-Mainly immune senescence or suppression.
			-Environmental and physiological factors may contribute
	Pain upon reactivation	-Not usually	-Nearly always neurological involvement
		-Some sensory loss with repeated recurrence	−90% of zoster has pain
			-May develop to post herpetic neuralgia

At the outset, the biological differences between VZV and HSV should be stressed, because they greatly affect the ability to study the viruses outside the human host. First, HSV-1 is robust for growth in tissue culture, while VZV is highly cell-associated, and cannot be obtained in titers needed to carry out synchronous VZV infections. Second, VZV is much more host restricted than HSV-1. There are numerous small animal models that accurately reflect primary infection, latency, and reactivation of HSV-1 in humans, but no animal model reproduces varicella, latency and zoster. Moreover, VZV is exceedingly difficult to experimentally reactivate from human ganglia. The lack of animal models has hampered studies of the adaptive immune response to VZV, which is problematic because human studies indicate cellular immune status is a major factor in VZV reactivation. For these reasons, our knowledge of mechanisms of latency and reactivation has advanced more rapidly for HSV than for VZV.

## The viral genomes

The proteomes of the two herpesviruses are quite similar. Most of the proteins encoded by each virus can be “matched”, based on conserved protein domains and amino acid sequence homologies. This includes a set of 41 “core” proteins found in all herpesviruses that encode basic critical functions, such as the key DNA replication proteins, capsid components and some enzymes. Genes with homology (orthologs) are arranged in the same order and direction in the genomes of both viruses (i.e. they are collinear). Only a handful of genes are virus specific: HSV-1 has 10 genes not present in VZV (latency associated transcripts, RL1 (ICP34.5), UL45, UL56, US2, US4 (glycoprotein G [gG]), US5(gJ), US6 (gD), US11, and US12 (ICP47): and all but six VZV ORFs have orthologs in the HSV genome (VZV unique genes are ORFs S/L, 1, 2, 13, 32, and 57). All VZV specific ORFs and all those in HSV-1 except gD can be deleted without preventing virus growth in culture, although most influence viral behavior and pathogenicity in animal models, as discussed below.

The DNA genomes of VZV and HSV show more difference at the sequence level. The HSV genome is larger (~152Kbp) than that of VZV (~125Kbp) and has, for unknown reasons, a much higher G + C content (67% for HSV, 47% for VZV). Both virus genomes can be divided into unique long and short regions, with each region being bounded by repeated sequences. However, the repeats of the HSV short region are 2.5 fold larger than those in VZV; and the HSV long region repeats are 9200 bp, compared to only 88.5 bp in VZV. The HSV genomes exist in virions as four linear isomers at equimolar ratios (involving inversion of the long and short regions with respect to each other), while VZV has two predominant isomers with a fixed orientation of the UL region packaged with two isomers of the short region. This is dictated, in part, by DNA packaging sequences located only in the left end of the VZV genome [3]. The repeat sequences encode duplicated genes, which for HSV include the multifunctional ICP0 regulatory protein, the LAT region, the γICP34.5 neurovirulence gene and the essential ICP4 transcriptional activator. Three VZV genes are duplicated in the short repeat regions, encoding IE62 (the VZV equivalent of HSV ICP4); IE63, which bears limited homology to ICP22 of HSV; and the ORF64 protein of poorly defined function. VZV has no apparent equivalent of the HSV LAT transcript or encode miRNAs [4]. This will be discussed in more detail in the section on latency.

## Viral protein function

Most HSV-1 protein functions have been determined by analyses of viral mutants. While VZV genetics has lagged behind HSV-1, over half of VZV genes have been analyzed in the background of VZV recombinants [5]. VZV protein functions that are not well understood are annotated based on knowledge of the HSV-1 counterpart. This must be taken with caution, as there is clear evolutionary divergence of the two viruses from a presumed common ancestor. While a full review of these differences is beyond the scope of this review, three illustrative examples are provided;

### Immediate early proteins

HSV encodes six immediate early proteins, five of which regulate gene expression, while VZV has only three reported IE genes. Both viruses encode an immediate early regulated transcriptional transactivator, (HSV-1 ICP4 and VZV ORF62) that is critical to virus replication [6,7] and shares sufficient functional similarity to enable VZV ORF62 to complement mutants of HSV-1 ICP4 [8,9]. However, VZV IE62 is a major virion tegument structural protein, while ICP4 is a minor virion protein [10]. The VZV ORF62 protein modulates the IFN response through alteration of the IFN signaling protein IRF3, an activity not yet reported for ICP4 [11]. While both viruses encode a protein with RNA post transcriptional processing activities (HSV-1 ICP27 and VZV ORF 4), the VZV ORF4 cannot complement HSV-1 ICP27 mutants [12]. In addition, both viruses encode proteins with E3 ubiquitin ligase activity (VZV ORF61 and HSV ICP0). However, HSV ICP0 disrupts ND10 bodies by degrading the ND10 components PML and SP100 and blocks IFN-γ signaling [13], whereas VZV ORF61 only partly modifies PML, does not fully disrupt ND10 bodies, and blocks IFN signaling less efficiently. Finally, VZV has no ortholog to the immune evasion ICP47, which modulates surface MHC-I antigen presentation for CD8 + T cell recognition [14].

### Short region proteins

The biggest difference in genomic organization of the two viruses lies in the short unique region, where VZV has only four proteins and HSV has 12. VZV lacks an equivalent to HSV-1 gD, which is found in all alpherpherpesviruses except VZV and its close cousin, simian varicella virus (SVV). gD is the HSV-1 receptor binding entry mediator, and binds the cellular receptors Herpesvirus entry mediator 1 and 2 (HVEM-1, HVEM 2) and Nectin-1 [15]. The obvious question is what mediates VZV cell binding and entry? This function is attributed to VZV gE, the most abundant VZV glycoprotein. Unlike the minor HSV-1 gE, VZV gE is essential for VZV infectivity. It has a novel specific N terminal domain that is important for binding to a VZV receptor, insulin degrading enzyme (ISG) [16-18]. While gE interacts with gI in both viruses, VZV gI is particularly critical in VZV neuroptropism [19,20].

### Tegument proteins

Both viruses encode many virion tegument proteins. A key illustrative divergence involves the cluster of 4 virion tegument proteins encoded by VZV ORFs 9–12 and HSV UL46-49. The HSV UL48 protein (also known as VP16), boosts IE gene expression by recruiting the host cell factors OCT1 and HCF to IE promoters, but is also critical for HSV-1 assembly and egress. In contrast, the VZV equivalent encoded by ORF10 is not needed for VZV IE gene expression or even for VZV growth in culture [21]. In contrast, HSV UL49 is not required for viral replication in culture whereas its VZV homolog (encoded by ORF9) is essential to VZV growth [22]. It is important to note that “non-essential” proteins are usually required for viral infection of organized tissues and animals models for each virus [23].

## Primary infection

Both VZV and HSV establish lytic infections at peripheral sites using very different routes. HSV-1 and HSV-2 usually initiate a primary infection at the periphery, and any subsequent recurrent infections following reactivation from latency occur at or near the same sites. HSV typically infects the oral cavity (HSV-1) or vaginal mucosa (HSV-2), but both viruses can infect at the skin, the eye, and other surfaces of the body. HSV infection rarely involves a systemic component except in infants or immunosuppressed patients. In mice, primary infection is restricted at least in part by type I and type II interferon responses, since abrogation of the respective receptors leads to increased systemic disease [24,25]. HSV replication is typically restricted to the epithelial layer of mucosal surfaces or the epidermal layer of the skin, particularly at sites of dermal abrasion. During the primary infection, virus gains access to the axonal termini of sensory neurons, is transported to neuronal nuclei in sensory ganglia and there establishes latency. HSV-1 replication at both the periphery and within sensory ganglia is largely controlled by the rapid deployment of an innate immune response, with subsequent involvement of adaptive immunity, as discussed below.

Unlike HSV, primary VZV infection involves a viremic phase and systemic delivery, in a manner more characteristic of infection with members of the Beta- and gammaherpesviruses. Elegant studies in the SCID-hu mouse model have contributed to a relatively new paradigm of VZV primary infection, in which inhalation of infectious virus initiates a primary infection at the respiratory lymphoid tissues, such as the tonsils [26,27]. There, VZV productively infects multiple circulatory cell types, including tonsilar CD4^+^T cells with an activated memory phenotype. VZV also infects professional antigen presenting cells such as monocytes, dendritic cells and macrophages [28-30], which may then transfer infectious virus to T cells at draining lymph nodes. The T cell associated viremia delivers virus systemically to skin and initiates sites of primary infection in the dermis following migration through the capillary vasculature. Thus, VZV infection initiates from the basal layer of the skin, whereas HSV initiates infection from the apical surface.

## Immunity to HSV-1 and VZV

HSV and VZV have formed synergistic relationships with their hosts that permit harmonious coexistence. The primary infection occurs in an immunologically naïve host and is controlled by an innate immune responses, followed by eventual development of adaptive immunity. However, recurrent HSV-1 and VZV disease is initiated in the face of a primed adaptive immune response, by a virus that is antigenically identical to that inducing immunity during the primary infection. The following sections briefly discuss the innate and adaptive immune responses to HSV-1 and VZV and the immune evasion mechanisms they employ to gain footholds in the primed host following reactivation from latency.

As our understanding of innate and adaptive immunity develops, the boundaries between the two systems have become increasingly fuzzy. We now know that innate and adaptive immunity cooperate to combat infections. However during primary infection, a temporal relationship exists in which the cells and molecules comprising the innate immune system have primary responsibility for controlling the virus, while the lymphocytes comprising the adaptive immune system undergo clonal expansion. A secondary response, orchestrated by lymphocytes of the adaptive immune system, often employs innate immune components as effector cells or molecules that lead to elimination of replicating virus.

### Innate immune control of HSV-1 and VZV

The innate immune system plays a critical role in controlling HSV-1 and VZV during the early stages of infection. Upon contact with an epithelial surface, the host recognizes pathogen-associated molecular patterns (PAMP) on the virus. Notable among these is the toll-like receptor (TLR) family, with an important role in activating innate immunity. The TLRs promote production of cytokines and other immunoregulatory molecules through intracellular signaling pathways, many employing the adaptor molecule MyD88, leading to nuclear translocation of nuclear factor (NF)-κB and transcription of genes encoding proinflammatory cytokines. TLR-2 and TLR-9 appear to be primarily responsible for recognition of HSV. Although the involvement of TLR in response to VZV is not well characterized, both type I and type II interferons are present in plasma of patients during the early incubation period when viral replication is thought to be mai contained primarily by innate immunity. Recent work has established an important role for TLR3 in preventing HSV induced encephalitis [31]. Given the similarity between HSV and VZV the involvement of TLR-2, -3, and −9 in controlling VZV is likely, but remains to be established.

The importance of interferons in combating both HSV and VZV infections is well established. HSV-1 infection of mice that are deficient in the interferon α/β and γ receptors, or in the signal transduction and transcription factor 1 (Stat1) are highly susceptible to disseminated HSV-1 infections [24,25]. Treatment of VZV infected SCID-hu mice with antibody to IFN-α/β results in a 10 fold increase in viral replication [32], mirroring the enhanced clearance of virus in immunocompromised children by type I interferon administration [33]. Plasmacytoid dendritic cells (pDC) are an important source of type I interferon during HSV-1 infections and are also attracted to VZV lesions, but in the latter case their capacity to produce type I interferon appears to be inhibited by VZV [28]. Nitric Oxide (NO) is another important effector molecule used by the innate immune system to combat HSV infections. Macrophages and inflammatory monocytes produce NO when infected with HSV in the presence of IFN-γ and TNF-α, and inhibition of NO increases HSV replication [34,35]. Finally, a novel innate defense mechanism recently reported for VZV is PML cages, which trap VZV nucleocapsids in some cell types. Of interest is that VZV does not degrade PML, as does is the case with HSV-1 [36].

### Adaptive T cell response to HSV and VZV

The HSV-1 murine model has enabled considerable assessments of the development of cellular immunity to infection. Following infection, dendritic cells (DC) carry HSV-1 to the draining lymph nodes for activation of CD4^+^ and CD8^+^ T cells. However, recent studies have established that initial expansion of HSV-specific CD8^+^ T cells in the draining lymph nodes of mice is induced by both lymph node resident CD8α^+^ DCs and by migratory DCs from the dermis [37-41]. Presumably the CD8α^+^ DCs cross-present viral antigens acquired from migratory DCs that originated at the site of infection or acquired from small amounts of free virus that reaches the lymph nodes through lymphatic vessels [42]. Both mature and immature DCs are susceptible to HSV infection [43-45], but only immature DCs support a productive infection and this leads to apoptosis [46]. HSV Infection of mature DCs results in an abortive infection in which HSV-1 α, β, and γ1 genes are expressed, but no infectious virions are produced [47]. VZV productively infects both immature and mature human DCs, and both HSV-1 and VZV infection of mature DC results in down-regulation of expression of MHC II and several costimulatory molecules resulting in impaired T cell stimulatory capacity [30,48]. Thus, DCs that are directly infected with either HSV-1 or VZV or those that phagocytose infected cells at the site of infection likely transport viral proteins to the regional lymph nodes. Those, that are directly infected would be inefficient at presenting them to T cells. HSV-specific CD4^+^ and CD8^+^ T cells are expanded following infection, and both populations invade infected skin and contribute to viral clearance [49]. Productive infection and immune evasion in DCs might play a role in the VZV viremic phase, either directly or by transfer to T cells in lymph nodes [29].

### Humoral immne response

HSV and VZV elicit a broad humoral response during infection. Antibodies are directed at viral glycoproteins, tegument or capsid proteins as well as other proteins [50,51]. These antibodies can neutralize the viruses. With VZV specific antibodies being more effective HSV elicits antibody responses including IgA and IgG [52]. VZV also elicits an antibodyÂ response including IgA, IgG, and IgM [53,54]. VZV specific IgG can be used to treat high-risk patients. HSV specific antibodies have been implicated in protection, but patients with lower antibody titers reactivate less, whereas people with higher levels of antibodies tend to reactivate more frequently [55,56].

### Immune evasion

Both viruses have evolved immune evasion mechanisms capable of blunting protective innate and adaptive immunity, which are thought to facilitate development of recurrent disease in the face of a robust immune response. The best characterized immune evasion molecule is the HSV immediate early protein ICP47 that binds to the Transporters A associated with Antigen Processing (TAPs) to inhibit peptide transport into the endoplasmic reticulum for loading onto MHC class I proteins. ICP47 presumably limits CD8^+^ T cell recognition of HSV infected cells, since the CD8^+^ T cell receptor can only recognize peptides bound to MHC class I. However the degree to which ICP47 activity contributes to pathogenesis is not clear. Even latently infected neurons with little HSV antigen or MHC I expression are clearly visible to CD8^+^ T cells since ganglionic CD8^+^ T cells display an activated phenotype in both mice and humans [57,58]. Moreover, CD8 + T cells can block HSV-1 reactivation from latency in mice and appear to play a major role in control of HSV-2 in the human genital tract [59]. This suggests that the effect of ICP47 can easily be overcome at sites of infection. Since IFN-γ can overcome the block of TAPs by ICP47, rapid IFN-γ production by NK cells at sites of infection might enhance recognition of infected cells by CD8^+^ T cells. VZV does not have an ICP47 homolog and indeed, does not block TAP peptide transport activity [60]. VZV does modulate surface expression of both MHC-I and MHC-II, with MHC-I being modulated in part by the ORF66 kinase [61,62]. HSV-1 also inhibits innate immunity through: (1) the virion host shut off protein, ICP0 and γ34.5, which inhibit type I interferon-mediated responses in infected cells; (2) glycoprotein C binding and inactivation of of C3b component of complement [63]; (3) an Fc binding ability of gE; and (4) through ICP22, Us5, Us3, and LAT, inhibition of NK cell and CD8 T cell apoptosis of HSV-1 infected cells [64]. VZV modulates IFN signaling, partly through the activity of the IE62 protein, which blocks IRF3 nuclear translocation [11]. The VZV IE63 also modulates IFN responses [65] and inhibits apoptosis [66]. VZV also blocks the NFkB signaling pathway in dendritic cells through activity of VZV ORF61 [67].

## Neuronal infection and the establishment of latency

Current knowledge of HSV neuronal latency is derived from multiple animal model systems that reproduce human latency, as well as from direct analyses of human ganglionic tissues. In contrast, our understanding of VZV latency is restricted by a lack of animal models and further complicated by the lower levels of latent VZV in human cadaver ganglionic tissues. Thus, apparent differences in HSV and VZV latency may, in part, reflect the different settings in which latency is studied. We know that both HSV-1 and VZV establish a persistent state predominantly in sensory neuronal nuclei. Consistent with the fact that most primary and recurrent HSV-1 infections occur in oral and nasal cavities or on the corneal surface without systemic spread, HSV-1 latency is most commonly observed in trigeminal ganglia (TG). In contrast, VZV DNA is distributed in sensory and autonomic ganglia across the entire neuraxis, consistent with the wide anatomical distribution of primary infection and reactivated disease can occur anywhere on the body. Quantitative estimates of viral genome copy numbers indicate VZV DNA is higher in the TG than in any single dorsal root ganglion [68] but is an order of magnitude lower than the HSV-1 latent load [69]. Both viruses access the ganglia by rretrograde axonal transport from the skin to neuronal nuclei, but VZV may also access nerve cell bodies by the hematogenous route during the viremic phase of infection. Thus, VZV has been found in ganglia such as the enteric ganglia that do not infiltrate the periphery [70]. The reduced latent VZV load could reflect factors such as age at primary infection, levels of HSV-1 and VZV exposure during primary infection, and the increased frequency of HSV-1 reactivation and shedding with increased genomic load at each recurrence.

## The maintenance of the HSV and VZV latent state

In human ganglia, both the VZV and HSV genomes appear as endless circular episomal structures that are associated with chromatin [1,68,71,72]. The heterochromatin state of the genomes is suspected to be responsible for the repression of gene expression and the productive cycle. However the limited transcription of both viruses during latency occurs in very different patterns.

HSV latency is relatively well characterized. In humans and in most animal models, latency is associated with the silencing of most viral gene transcription and the abundant expression of Latency Associated Transcripts (LATs) in some latently infected neurons. LATs lie partly complementary to the 3′ end of the ICP0 gene and are stable nuclear RNAs without polyadenylation or 5′ caps that have structural features of stable lariats of introns. They do not encode protein, and may be byproducts of a larger unstable primary transcript that is processed into microRNAs [4,73]. Although viral mutants lacking LATs are able to establish, maintain, and reactivate from latency; LATs do appear to increase the efficiency of latency establishment and of reactivation. LATs may boost latency establishment through their ability to block neuronal apoptosis, thus increasing the survival of latently infected neurons [74]. Moreover, several HSV-1 microRNAs RNAs (miRNAs) that localize to the LAT transcript [73] may inhibit viral lytic gene expression during latency, though an exact mechanism remains undefined. A recent study has suggested that miRNAs regulating the neruovirulence factor γ34.5 may contribute to latency and virulence [75]. LATs also influence by as yet unresolved mechanisms the preferential retention of latent HSV-1 and HSV-2 in the A5 and KH10 antibody marked neuronal subtypes, respectively, in mice [76].

Emerging evidence demonstrates that latent HSV-1 is not a silent infection that is ignored by host immunity. Nested PCR and in situ hybridization approaches suggest low level RNA expression of some viral lytic genes during HSV-1 latency in murine sensory ganglia [77,78], and sporadic expression of proteins in rare neurons was observed [78]. Several groups have now demonstrated that HSV-specific CD8^+^ T cells infiltrate murine sensory ganglia during acute HSV-1 infection, and remain in direct apposition to the neurons during latency. These ganglion-resident CD8+ T cells exhibit an activated phenotype and polarize their antigen receptors to form an “immunological synapse” with neurons, demonstrating recognition of HSV antigens in context of MHC-I [79,80]. The retention of HSV-specific CD8^+^ T cells in latently infected TG requires recognition of viral peptides, since the vast majority of CD8^+^ T cells in latently infected murine TG are HSV-specific, and CD8^+^ T cells of irrelevant specificity are lost from the latently infected ganglia over time [81-83]. CD8^+^ T cells with a similar activation phenotype have also been observed in direct apposition to HSV-1 latently infected neurons in human TG [57,58]. Taken together, these studies strongly suggest an occasional release of HSV-1 protein repression during HSV-1 latency, though the structural and functional integrity of these viral proteins is uncertain.

VZV appears to lack an equivalent of the LAT transcript, but does express some lytic transcripts during latency. Several VZV lytic transcripts have been observed in ganglia of VZV infected rodents that do not support VZV replication. However, such expression could reflect an abortive infection, as VZV does not replicate or reactivate in the animal models. Latent gene expression has been reported in guinea pigs, the only small immune competent animal model in which VZV replicates. Latent VZV gene expression has not been extensively analyzed in this model, although a report has suggested VZV can be reactivated from persistently infected guinea pig enteric neurons [70]. VZV enters a persistent state in SCID-hu mice harboring human ganglionic tissue despite the inability of these mice to mount an adaptive immune response [84]. The ganglia of these mice show expression of ORF63 RNA, but little detectable protein. New in vitro models of VZV latency resulting from infection of explanted human ganglia [85] or of human stem cell-derived neurons [86] should enhance our knowledge of latency, although such models lack the critical aspect of immunity, which is an established factor in maintenance of the VZV latent state.

Much of the current understanding of VZV latency-associated transcription comes from analyses of human cadaver ganglia. Several viral lytic RNAs and even proteins have been reported to be expressed in VZV latently infected dorsal root ganglia [69,87-89], including from VZV ORFs 4, 21, 61, 10, 29, 62, 63 and 66 [84,88,90-98]. The ORF most commonly and abundantly detected during VZV latency is ORF63 [84,94,99], a protein that modulates the activity of type I IFNs [65,100,101] and affects apoptosis in neurons. Reports of VZV protein expression during latency in human ganglia [95,102-104], have been put into question by the recent demonstration that many antibodies used in these studies show cross reactivity to blood group A antigensÂ (123). If VZV proteins are made during latency, the question arises as to how neurons expressing VZV protein antigens avoid immune elimination. The possible involvement of VZV immune evasion mechanisms will be discussed below. The validity of transcripts seen VZV latent state has also recently been put into some question, since it appears the VZV genome undergoes post mortem transcription (124). Thus, VZV latency remains in an unresolved and confusing state.

## HSV Vs VZV reactivation from latency and recrudescent disease

Any discussion of HSV-1 and VZV reactivation from latency must be prefaced by a carful definition of terms. One can define latency as retention of viral genome in the nucleus of a cell for an extended period without production of infectious virions. Reactivation would then be the point at which virion formation occurs, which would permit limited viral gene expression without reactivation. From a molecular virological standpoint, one might define latency as retention of the viral genome in the nucleus of a cell for an extended period without any viral gene expression (except perhaps for the HSV LAT gene). By this definition, reactivation occurs at the point of initiation of viral gene expression. For the purpose of this discussion we define reactivation as production of infectious virus following a period of latency. The epidemiology and pathophysiology of HSV and VZV recurrent disease suggest important differences in the way the two viruses reactivate from latency.

While both viruses establish latent infections in sensory neurons, the manner in which the two viruses reactivate and the recrudescent disease they cause is quite different. HSV-1 reactivates frequently and can produce clinically apparent lesions throughout life, but recurrence becomes less frequent with age. In contrast, Herpes zoster typically occurs only once after age 50, with incidence rising with age and declining cellular immune status. Secondly, reactivation of HSV rarely involves more than one or a few neurons, while Herpes zoster reflects peripheral delivery of HSV vs VZV from many neurons. There is little, if any, interneuronal spread of HSV-1 prior to anterograde axonal transport to the peripheral site, possibly reflecting local immune control or the separate transport of viral capsids and envelop proteins for assembly of the infectious virus at the peripheral nerve termini [105,106]. In contrast, the large lesions of zoster reflect peripheral delivery by multiple neurons originating from the host ganglia, and there is obvious extensive intraganglionic spread of reactivating VZV prior to peripheral delivery. The virus may also spread centrally to induce CNS disease. The different pattern of VZV disease likely reflects infectious virus assembly at the ganglia and/or a lack of immunity to control the spread.

Subclinical reactivation and shedding of HSV occurs relatively frequently, though the factors that determine if HSV shedding results in lesion formation remain largely obscure. While subclinical reactivation and shedding of VZV is not as well studied, can occur, as demonstrated VZV shedding in saliva of astronauts under conditions of extreme stress [107]. VZV can cause pain and neurological disease without skin rash [108]. HSV-1 reactivation is triggered by a variety of factors, either environmental (stress and UV-B irradiation) or physiologic factors (changes in female sex hormones) that do not appear to be sufficient to trigger VZV reactivation [109,110]. The triggers of HSV-1 reactivation are all associated with transient inhibition of T cell function, and growing evidence supports a role for CD8^+^ T cells in preventing HSV-1 reactivation from latency in sensory neurons. Ganglionic CD8^+^ T cells retained during latency in direct apposition to infected TG neurons in C57BL/6 mice are HSV-1 specific [81], release the effector molecules interferon gamma (IFN-γ) and lytic granules when stimulated directly ex vivo, and can block HSV-1 reactivation from latency in sensory neurons [79,80,111]. Interestingly, lytic granule release into infected neurons does not lead to neuronal apoptosis [112], possibly due to the ability of LATs to inhibit granzyme B activation of the neuronal caspase system [112,113]. Instead, granzyme B appears to block reactivation at least in part through cleavage of the critical HSV-1 regulatory protein ICP4 [112]. Collectively these findings suggest that reactivation from HSV-1 latency is prevented by the constant vigilance of CD8^+^ T cells that surround latently infected neurons, and that factors that transiently compromise their function allows the virus to escape immune control [109,110,114].

Cellular immunity clearly plays a critical role in controlling VZV reactivation, because the two main factors contributing to zoster incidence are age (most likely reflecting immune senescence) and cellular immune compromise due to disease or iatrogenic cause. However the nature of that response is not at all clear. The apparent important role for CD8^+^ T cells in maintaining HSV-1 latency [79,80,112], suggests their possible involvement in maintaining VZV latency. However, VZV specific T cell infiltrates have not been described in VZV latently infected human ganglia. The fact that shingles/zoster typically occurs only once is thought to be related to bolstering of the flagging immune system by re-exposure to viral antigens, a notion that is supported by the observations of multiple occurrences in immunosuppressed patients, and the success of the zoster vaccine in reducing the occurrence of zoster/shingles [115].

The molecular mechanisms that maintain HSV-1 and VZV latency and how they are overcome during reactivation is not clear. This is particularly true for VZV, which has never been experimentally reactivated (with two possible exceptions [70,97]). For HSV-1, it is clear that the chromatin state of the viral genome is important for repression. A model scenario of HSV-1 reactivation is that both heterochromatin mediated repression and immunity must be overcome for HSV reactivation and virus delivery at the periphery. Delineation of the mechanisms that regulate the chromatin state of the viral genome is rapidly progressing [72]. For example, signal transduction in cultured neurons may lead to transient gene expression through chromatin alternation, which then may progress to a normal lytic cycle if unchecked [116]. Others have proposed the switch between latency and release of gene expression is dependent upon cellular localization of HSV IE gene activator HCF and its response to signals to the host neuron [117]. For VZV, this is an almost completely unexplored area.

An important issue for reactivation is pain, which is regularly associated with VZV and rarely associated with HSV-1. Over 30% of zoster cases develop a chronic neuropathic pain state after resolution of visible skin disease, referred to as post herpetic neuralgia [PHN]. While PHN appears to have multiple causes, it is clear that a major contributing factor is the extensive insult of ganglionic VZV replication, intraganglionic spread and resulting inflammation and altered neuronal physiology. The involvement of many sensory neurons likely amplifies any virus induced changes to neurons that may contribute to pain. Ganglionic inflammatory responses may also contribute to pain, since in some cases PHN symptoms are alleviated by steroid treatment [118]. A recent study has shown that following zoster, there is extensive monocyte and T cell infiltration of reactivating ganglia [119]. However, it is unclear if these cells exacerbate or alleviate pain. The fact that the zoster vaccine reduces the severity of VZV disease and PHN indicates suggests a palliative role for adaptive immunity in PHN. A new model of VZV induced pain in immune competent rats [120,121] may provide a better understanding of the contributions of host immunity to VZV induced pain.

Primary and recrudescent HSV-1 lesions are not associated with significant pain, even though HSV-1 acutely and latently infected TG display a significant leukocytic infiltrate. Clearly, the involvement of only a single or few neurons limits any physiological changes, but the nature of the inflammatory infiltrate might also be important in determining if infection of sensory neurons is associated with pain. The infiltrate in human and murine latently infected TG is comprised exclusively of mononuclear cells with a preponderance of CD4^+^ and CD8^+^ T cells and macrophages. The composition does not appear to change appreciably following stress-induced reactivation. Although the CD8^+^ T cells that localize to infected neurons are persistently activated and release their effector molecules during the course of immunosurvellance, this does not appear to induce a pain response in neurons [57,58,112].

## Conclusion

It emerges from the above discussion that HSV and VZV, while sharing many structural and molecular features, are pathophysiologically unique. The genomic similarities, biphasic lytic/latent life cycles, and selective establishment of latency in sensory neurons, are contrasted by the very different patterns of primary infection and reactivated disease. The most striking differences between the two viruses include: 1) the broader host range of HSV; 2) the route of infection and spread; 3) possible differences in the route of transport to the sensory neurons (strictly neurologic route for HSV-1, possibly both neurologic and hematogenous route for VZV); 4) production of LATs only by HSV; 5) possible lytic gene expressionÂ by VZV during latency; 6) reduced susceptibility of VZV to environmental and physiologic reactivation stimuli; 7) area of involvement in recrudescent disease; and 8) the pain uniquely associated with VZV reactivation. Additional similarities and differences between the two viruses might emerge as better animal models of VZV infection and latency are developed. While great progress has been made in understanding the biology of the two viruses, many questions remain. What are the mechanisms by which HSV-1 and VZV establish latent infections in sensory neurons and why are the reactivation patterns so different? Why do systemic stimuli (hyperthermia, stress, etc.) induce HSV-1 reactivation in only one or a few neurons despite many thousands of neurons harboring latent virus? Most importantly, why does an effective HSV vaccine remain elusive despite the volumes of data describing the immune response to HSV, while the VZV vaccine is so effective it has completely changed the epidemiology of primary VZV disease in the USA?

Answers to some of these questions may already be emerging. For instance, the demonstration of a role for CD8^+^ T cells in preventing HSV-1 reactivation and spread of HSV-1 in the genital tract might suggest that vaccines targeting CD8^+^ T cells will exhibit improved efficacy. Also, it was recently established that 80% of HSV-specific CD8^+^ T cells in C57BL/6 mice are reactive to viral proteins that are produced before viral DNA synthesis, and a very high percentage target three viral proteins, glycoprotein B (gB), ribonucleotide reductase 1 (RR1), and ICP8. Since mice establish latent HSV infections, but do not spontaneously reactivate the virus, they may represent a model system in which immune control of the viral latency is optimized. Therefore, a vaccine that targets human T cells that are reactive to non-structural, non-surface antigens as well as the usual glycoproteins might prove effective. However, the apparent sequestration of the TG-resident CD8^+^ T cell population is an obstacle that will need to be overcome in any vaccine strategy designed to bolster the CD8^+^ T cell immunesurveillance of latently infected neurons [114,122]. The broader neuronal involvement in VZV reactivation might reflect an apparent failure of VZV latently infected neurons to attract T cells and macrophages prior to reactivation [119]. The T cells and macrophages that are juxtaposed to HSV-1 latently infected neurons might rapidly restrict the lateral spread of reactivated virus within the ganglion, thus reducing the size of the involved dermatome. HSV research continues on a very steep trajectory. While VZV research has been hampered by a lack of animal models, recent studies in hu-SCID mice and other animal models appear very promising.

## Competing interests

The authors declare that they have no competing interests.

## Authors’ contributions

PRK was the main contributor to the manuscript; RLH, AJSL, and J-MG provided portions of the manuscript and participated in the revisions and editing of the manuscript. All authors read and approved the final manuscript.

## References

[B1] Mitchell BM Bloom DC Cohrs RJ Gilden DH Kennedy PG Herpes simplex virus-1 and varicella-zoster virus latency in ganglia J Neurovirol 2003 9 194 204 12707850 10.1080/13550280390194000

[B2] Rouse BT Kaistha SD A tale of 2 alpha-herpesviruses: lessons for vaccinologists Clin Infect Dis 2006 42 810 817 10.1086/500141 16477558

[B3] Kaufer BB Smejkal B Osterrieder N The varicella-zoster virus ORFS/L (ORF0) gene is required for efficient viral replication and contains an element involved in DNA cleavage J Virol 2010 84 11661 11669 10.1128/JVI.00878-10 20844039 PMC2977885

[B4] Umbach JL Nagel MA Cohrs RJ Gilden DH Cullen BR Analysis of human alphaherpesvirus microRNA expression in latently infected human trigeminal ganglia J Virol 2009 83 10677 10683 10.1128/JVI.01185-09 19656888 PMC2753103

[B5] Cohen JI The varicella-zoster virus genome Curr Top Microbiol Immunol 2010 342 1 14 10.1007/82_2010_10 20225013 PMC3413377

[B6] DeLuca NA McCarthy AM Schaffer PA Isolation and characterization of deletion mutants of herpes simplex virus type 1 in the gene encoding immediate-early regulatory protein ICP4 J Virol 1985 56 558 570 2997476 10.1128/jvi.56.2.558-570.1985 PMC252613

[B7] Sato B Ito H Hinchliffe S Sommer MH Zerboni L Arvin AM Mutational analysis of open reading frames 62 and 71, encoding the varicella-zoster virus immediate-early transactivating protein, IE62, and effects on replication in vitro and in skin xenografts in the SCID-hu mouse in vivo J Virol 2003 77 5607 5620 10.1128/JVI.77.10.5607-5620.2003 12719553 PMC154054

[B8] Felser JM Kinchington PR Inchauspe G Straus SE Ostrove JM Cell lines containing varicella-zoster virus open reading frame 62 and expressing the “IE” 175 protein complement ICP4 mutants of herpes simplex virus type 1 J Virol 1988 62 2076 2082 2835512 10.1128/jvi.62.6.2076-2082.1988 PMC253298

[B9] Tyler JK Orr A Everett RD Replacement of the herpes simplex virus type 1 Vmw175 DNA binding domain with its varicella-zoster virus counterpart results in a protein with novel regulatory properties that can support virus growth J Gen Virol 1997 78 Pt 1 179 188 9010302 10.1099/0022-1317-78-1-179

[B10] Kinchington PR Fite K Seman A Turse SE Virion association of IE62, the varicella-zoster virus (VZV) major transcriptional regulatory protein, requires expression of the VZV open reading frame 66 protein kinase J Virol 2001 75 9106 9113 10.1128/JVI.75.19.9106-9113.2001 11533174 PMC114479

[B11] Sen N Sommer M Che X White K Ruyechan WT Arvin AM Varicella-zoster virus immediate-early protein 62 blocks interferon regulatory factor 3 (IRF3) phosphorylation at key serine residues: a novel mechanism of IRF3 inhibition among herpesviruses J Virol 2010 84 9240 9253 10.1128/JVI.01147-10 20631144 PMC2937611

[B12] Perera LP Kaushal S Kinchington PR Mosca JD Hayward GS Straus SE Varicella-zoster virus open reading frame 4 encodes a transcriptional activator that is functionally distinct from that of herpes simplex virus homology ICP27 J Virol 1994 68 2468 2477 8139031 10.1128/jvi.68.4.2468-2477.1994 PMC236724

[B13] Kyratsous CA Walters MS Panagiotidis CA Silverstein SJ Complementation of a herpes simplex virus ICP0 null mutant by varicella-zoster virus ORF61p J Virol 2009 83 10637 10643 10.1128/JVI.01144-09 19656893 PMC2753114

[B14] Hill A Jugovic P York I Russ G Bennink J Yewdell J Ploegh H Johnson D Herpes simplex virus turns off the TAP to evade host immunity Nature 1995 375 6530 411 415 10.1038/375411a0 7760935

[B15] Spear PG Herpes simplex virus: receptors and ligands for cell entry Cell Microbiol 2004 6 401 410 10.1111/j.1462-5822.2004.00389.x 15056211

[B16] Berarducci B Rajamani J Zerboni L Che X Sommer M Arvin AM Functions of the unique N-terminal region of glycoprotein E in the pathogenesis of varicella-zoster virus infection Proc Natl Acad Sci U S A 2010 107 282 287 10.1073/pnas.0912373107 19966293 PMC2806775

[B17] Li Q Krogmann T Ali MA Tang WJ Cohen JI The amino terminus of varicella-zoster virus (VZV) glycoprotein E is required for binding to insulin-degrading enzyme, a VZV receptor J Virol 2007 81 8525 8532 10.1128/JVI.00286-07 17553876 PMC1951364

[B18] Li Q Ali MA Cohen JI Insulin degrading enzyme is a cellular receptor mediating varicella-zoster virus infection and cell-to-cell spread Cell 2006 127 305 316 10.1016/j.cell.2006.08.046 17055432 PMC7125743

[B19] Oliver SL Sommer MH Reichelt M Rajamani J Vlaycheva-Beisheim L Stamatis S Cheng J Jones C Zehnder J Arvin AM Mutagenesis of varicella-zoster virus glycoprotein I (gI) identifies a cysteine residue critical for gE/gI heterodimer formation, gI structure, and virulence in skin cells J Virol 2011 85 4095 4110 10.1128/JVI.02596-10 21345964 PMC3126246

[B20] Arvin AM Oliver S Reichelt M Moffat JF Sommer M Zerboni L Berarducci B Analysis of the functions of glycoproteins E and I and their promoters during VZV replication in vitro and in skin and T-cell xenografts in the SCID mouse model of VZV pathogenesis Curr Top Microbiol Immunol 2010 342 129 146 10.1007/82_2009_1 20186616

[B21] Cohen JI Seidel K Varicella-zoster virus (VZV) open reading frame 10 protein, the homolog of the essential herpes simplex virus protein VP16, is dispensable for VZV replication in vitro J Virol 1994 68 7850 7858 7966575 10.1128/jvi.68.12.7850-7858.1994 PMC237247

[B22] Tischer BK Kaufer BB Sommer M Wussow F Arvin AM Osterrieder N A self-excisable infectious bacterial artificial chromosome clone of varicella-zoster virus allows analysis of the essential tegument protein encoded by ORF9 J Virol 2007 81 13200 13208 10.1128/JVI.01148-07 17913822 PMC2169085

[B23] Che X Reichelt M Sommer MH Rajamani J Zerboni L Arvin AM Functions of the ORF9-to-ORF12 gene cluster in varicella-zoster virus replication and in the pathogenesis of skin infection J Virol 2008 82 5825 5834 10.1128/JVI.00303-08 18400847 PMC2395146

[B24] Pasieka TJ Lu B Leib DA Enhanced Pathogenesis of an Attenuated Herpes Simplex Virus for Mice Lacking Stat1 J Virol 2008 82 6052 6055 10.1128/JVI.00297-08 18400863 PMC2395151

[B25] Leib DA Harrison TE Laslo KM Machalek MA Moorman NJ Virgin HW Interferons Regulate the Phenotype of Wild-type and Mutant Herpes Simplex Viruses In Vivo J Exp Med 1999 189 663 672 10.1084/jem.189.4.663 9989981 PMC2192939

[B26] Arvin AM Moffat JF Sommer M Oliver S Che X Vleck S Zerboni L Ku CC Varicella-zoster virus T cell tropism and the pathogenesis of skin infection Curr Top Microbiol Immunol 2010 342 189 209 10.1007/82_2010_29 20397071 PMC4077053

[B27] Ku CC Besser J Abendroth A Grose C Arvin AM Varicella-Zoster virus pathogenesis and immunobiology: new concepts emerging from investigations with the SCIDhu mouse model J Virol 2005 79 2651 2658 10.1128/JVI.79.5.2651-2658.2005 15708984 PMC548427

[B28] Huch JH Cunningham AL Arvin AM Nasr N Santegoets SJ Slobedman E Slobedman B Abendroth A Impact of varicella zoster virus on dendritic cell subsets in human skin during natural infection J Virol 2010 84 4060 4072 10.1128/JVI.01450-09 20130046 PMC2849518

[B29] Morrow G Slobedman B Cunningham AL Abendroth A Varicella-zoster virus productively infects mature dendritic cells and alters their immune function J Virol 2003 77 4950 4959 10.1128/JVI.77.8.4950-4959.2003 12663800 PMC152143

[B30] Abendroth A Morrow G Cunningham AL Slobedman B Varicella-zoster virus infection of human dendritic cells and transmission to T cells: implications for virus dissemination in the host J Virol 2001 75 6183 6192 10.1128/JVI.75.13.6183-6192.2001 11390620 PMC114334

[B31] Guo Y Audry M Ciancanelli M Alsina L Azevedo J Herman M Anguiano E Sancho-Shimizu V Lorenzo L Pauwels E Herpes simplex virus encephalitis in a patient with complete TLR3 deficiency: TLR3 is otherwise redundant in protective immunity J Exp Med 2011 208 2083 2098 10.1084/jem.20101568 21911422 PMC3182056

[B32] Ku C-C Zerboni L Ito H Graham BS Wallace M Arvin AM Varicella-Zoster Virus Transfer to Skin by T Cells and Modulation of Viral Replication by Epidermal Cell Interferon-α J Exp Med 2004 200 917 925 10.1084/jem.20040634 15452178 PMC2213285

[B33] Arvin AM Kushner JH Feldman S Baehner RL Hammond D Merigan TC Human Leukocyte Interferon for the Treatment of Varicella in Children with Cancer N Engl J Med 1982 306 761 765 10.1056/NEJM198204013061301 6174864

[B34] Baskin H Ellermann-Eriksen S Lovmand J Mogensen SC Herpes simplex virus type 2 synergizes with interferon-gamma in the induction of nitric oxide production in mouse macrophages through autocrine secretion of tumour necrosis factor-alpha J Gen Virol 1997 78 195 203 9010304 10.1099/0022-1317-78-1-195

[B35] Kodukula P Liu T Rooijen NV Jager MJ Hendricks RL Macrophage Control of Herpes Simplex Virus Type 1 Replication in the Peripheral Nervous System J Immunol 1999 162 2895 2905 10072539

[B36] Reichelt M Wang L Sommer M Perrino J Nour AM Sen N Baiker A Zerboni L Arvin AM Entrapment of Viral Capsids in Nuclear PML Cages Is an Intrinsic Antiviral Host Defense against Varicella-Zoster Virus PLoS Pathog 2011 7 e1001266 10.1371/journal.ppat.1001266 21304940 PMC3033373

[B37] Allan RS Smith CM Belz GT van Lint AL Wakim LM Heath WR Carbone FR Epidermal Viral Immunity Induced by CD8{Abendroth, #49}+Dendritic Cells But Not by Langerhans Cells Science 2003 301 1925 1928 10.1126/science.1087576 14512632

[B38] Allan RS Waithman J Bedoui S Jones CM Villadangos JA Zhan Y Lew AM Shortman K Heath WR Carbone FR Migratory Dendritic Cells Transfer Antigen to a Lymph Node-Resident Dendritic Cell Population for Efficient CTL Priming Immunity 2006 25 153 162 10.1016/j.immuni.2006.04.017 16860764

[B39] Jirmo AC Nagel C-H Bohnen C Sodeik B Behrens GMN Contribution of Direct and Cross-Presentation to CTL Immunity against Herpes Simplex Virus 1 J Immunol 2009 182 283 292 19109159 10.4049/jimmunol.182.1.283

[B40] Smith CM Belz GT Wilson NS Villadangos JA Shortman K Carbone FR Heath WR Cutting Edge: Conventional CD8α + Dendritic Cells Are Preferentially Involved in CTL Priming After Footpad Infection with Herpes Simplex Virus-1 J Immunol 2003 170 4437 4440 12707318 10.4049/jimmunol.170.9.4437

[B41] Lee HK Zamora M Linehan MM Iijima N Gonzalez D Haberman A Iwasaki A Differential roles of migratory and resident DCs in T cell priming after mucosal or skin HSV-1 infection J Exp Med 2009 206 359 370 10.1084/jem.20080601 19153243 PMC2646574

[B42] Sprecher E Becker Y Langerhans cell density and activity in mouse skin and lymph nodes affect herpes simplex type 1 (HSV-1) pathogenicity Arch Virol 1989 107 191 205 10.1007/BF01317916 2554853

[B43] Geraghty RJ Krummenacher C Cohen GH Eisenberg RJ Spear PG Entry of Alphaherpesviruses Mediated by Poliovirus Receptor-Related Protein 1 and Poliovirus Receptor Science 1998 280 1618 1620 10.1126/science.280.5369.1618 9616127

[B44] Montgomery RI Warner MS Lum BJ Spear PG Herpes Simplex Virus-1 Entry into Cells Mediated by a Novel Member of the TNF/NGF Receptor Family Cell 1996 87 427 436 10.1016/S0092-8674(00)81363-X 8898196

[B45] Chiu YG Bowers WJ Lim ST Ryan DA Federoff HJ Effects of Herpes Simplex Virus Amplicon Transduction on Murine Dendritic Cells Hum Gene Ther 2009 20 442 452 10.1089/hum.2008.160 19199821 PMC2828628

[B46] Mikloska Z Bosnjak L Cunningham AL Immature Monocyte-Derived Dendritic Cells Are Productively Infected with Herpes Simplex Virus Type 1 J Virol 2001 75 5958 5964 10.1128/JVI.75.13.5958-5964.2001 11390597 PMC114311

[B47] Kruse M Rosorius O Kratzer F Stelz G Kuhnt C Schuler G Hauber J Steinkasserer A Mature Dendritic Cells Infected with Herpes Simplex Virus Type 1 Exhibit Inhibited T-Cell Stimulatory Capacity J Virol 2000 74 7127 7136 10.1128/JVI.74.15.7127-7136.2000 10888653 PMC112231

[B48] Morrow G Slobedman B Cunningham AL Abendroth A Varicella-Zoster Virus Productively Infects Mature Dendritic Cells and Alters Their Immune Function J Virol 2003 77 4950 4959 10.1128/JVI.77.8.4950-4959.2003 12663800 PMC152143

[B49] Rajasagi NK Kassim SH Kollias CM Zhao X Chervenak R Jennings SR CD4+ T Cells Are Required for the Priming of CD8+ T Cells following Infection with Herpes Simplex Virus Type 1 J Virol 2009 83 5256 5268 10.1128/JVI.01997-08 19279095 PMC2682109

[B50] Ashley R Wald A Corey L Cervical antibodies in patients with oral herpes simplex virus type 1 (HSV-1) infection: local anamnestic responses after genital HSV-2 infection J Virol 1994 68 5284 5286 8035526 10.1128/jvi.68.8.5284-5286.1994 PMC236475

[B51] Ashley R Benedetti J Corey L Humoral immune response to HSV-1 and HSV-2 viral proteins in patients with primary genital herpes J Med Virol 1985 17 153 166 10.1002/jmv.1890170208 2997384

[B52] Russell MW Mestecky J Humoral immune responses to microbial infections in the genital tract Microbes Infect 2002 4 667 677 10.1016/S1286-4579(02)01585-X 12048036

[B53] Bogger-Goren S Baba K Hurley P Yabuuchi H Takahashi M Ogra PL Antibody response to varicella-zoster virus after natural or vaccine-induced infection J Infect Dis 1982 146 260 265 10.1093/infdis/146.2.260 6286790

[B54] Brunell PA Gershon AA Uduman SA Steinberg S Varicella-Zoster Immunoglobulins during Varicella, Latency, and Zoster J Infect Dis 1975 132 49 54 10.1093/infdis/132.1.49 169308

[B55] Spruance SL Evans TG McKeough MB Thai L Araneo BA Daynes RA Mishkin EM Abramovitz AS Th1/Th2-like immunity and resistance to herpes simplex labialis Antivir Res 1995 28 39 55 10.1016/0166-3542(95)00037-M 8585759

[B56] Koelle DM Corey L Herpes Simplex: Insights on Pathogenesis and Possible Vaccines Annu Rev Med 2008 59 381 395 10.1146/annurev.med.59.061606.095540 18186706

[B57] Theil D Derfuss T Paripovic I Herberger S Meinl E Schueler O Strupp M Arbusow V Brandt T Latent Herpesvirus Infection in Human Trigeminal Ganglia Causes Chronic Immune Response Am J Pathol 2003 163 2179 2184 10.1016/S0002-9440(10)63575-4 14633592 PMC1892378

[B58] Verjans GMGM Hintzen RQ van Dun JM Poot A Milikan JC Laman JD Langerak AW Kinchington PR Osterhaus ADME Selective retention of herpes simplex virus-specific T cells in latently infected human trigeminal ganglia Proc Natl Acad Sci 2007 104 3496 3501 10.1073/pnas.0610847104 17360672 PMC1805572

[B59] Zhu J Koelle DM Cao J Vazquez J Huang ML Hladik F Wald A Corey L Virus-specific CD8+ T cells accumulate near sensory nerve endings in genital skin during subclinical HSV-2 reactivation J Exp Med 2007 204 595 603 10.1084/jem.20061792 17325200 PMC2137910

[B60] Verweij MC Lipinska AD Koppers-Lalic D van Leeuwen WF Cohen JI Kinchington PR Messaoudi I Bienkowska-Szewczyk K Ressing ME Rijsewijk FAM Wiertz EJHJ The Capacity of UL49.5 Proteins To Inhibit TAP Is Widely Distributed among Members of the Genus Varicellovirus J Virol 2011 85 2351 2363 10.1128/JVI.01621-10 21159875 PMC3067808

[B61] Eisfeld AJ Yee MB Erazo A Abendroth A Kinchington PR Downregulation of Class I Major Histocompatibility Complex Surface Expression by Varicella-Zoster Virus Involves Open Reading Frame 66 Protein Kinase-Dependent and -Independent Mechanisms J Virol 2007 81 9034 9049 10.1128/JVI.00711-07 17567702 PMC1951447

[B62] Abendroth A Kinchington PR Slobedman B Varicella zoster virus immune evasion strategies Curr Top Microbiol Immunol 2010 342 155 171 10.1007/82_2010_41 20563710 PMC3936337

[B63] Friedman HM Cohen GH Eisenberg RJ Seidel CA Cines DB Glycoprotein C of herpes simplex virus 1 acts as a receptor for the C3b complement component on infected cells Nature 1984 309 633 635 10.1038/309633a0 6328323

[B64] Aubert M O’Toole J Blaho JA Induction and Prevention of Apoptosis in Human HEp-2 Cells by Herpes Simplex Virus Type 1 J Virol 1999 73 10359 10370 10559354 10.1128/jvi.73.12.10359-10370.1999 PMC113091

[B65] Ambagala AP Cohen JI Varicella-Zoster virus IE63, a major viral latency protein, is required to inhibit the alpha interferon-induced antiviral response J Virol 2007 81 7844 7851 10.1128/JVI.00325-07 17507475 PMC1951283

[B66] Hood C Cunningham AL Slobedman B Arvin AM Sommer MH Kinchington PR Abendroth A Varicella-Zoster Virus ORF63 Inhibits Apoptosis of Primary Human Neurons J Virol 2006 80 1025 1031 10.1128/JVI.80.2.1025-1031.2006 16379003 PMC1346839

[B67] Sloan E Henriquez R Kinchington P Slobedman B Abendroth A Varicella zoster virus inhibition of the NFκB pathway during infection of human dendritic cells: role for ORF61 as a modulator of NFκB activity J Virol 2011 86 1193 1202 22090112 10.1128/JVI.06400-11 PMC3255823

[B68] Azarkh Y Gilden D Cohrs RJ Molecular characterization of varicella zoster virus in latently infected human ganglia: physical state and abundance of VZV DNA, Quantitation of viral transcripts and detection of VZV-specific proteins Curr Top Microbiol Immunol 2010 342 229 241 10.1007/82_2009_2 20186615 PMC3016873

[B69] Pevenstein SR Williams RK McChesney D Mont EK Smialek JE Straus SE Quantitation of latent varicella-zoster virus and herpes simplex virus genomes in human trigeminal ganglia J Virol 1999 73 10514 10518 10559370 10.1128/jvi.73.12.10514-10518.1999 PMC113107

[B70] Gershon AA Chen J Gershon MD A model of lytic, latent, and reactivating varicella-zoster virus infections in isolated enteric neurons J Infect Dis 2008 197 Suppl 2 S61 S65 18419411 10.1086/522149

[B71] Gary L Gilden DH Cohrs RJ Epigenetic regulation of varicella-zoster virus open reading frames 62 and 63 in latently infected human trigeminal ganglia J Virol 2006 80 4921 4926 10.1128/JVI.80.10.4921-4926.2006 16641283 PMC1472082

[B72] Knipe DM Howley PM Griffin DE Fields’ Virology 2007 5 Philadelphia: Wolters Kluwer Health/Lippincott Williams & Wilkins 2503-2577 2775-2807

[B73] Umbach JL Kramer MF Jurak I Karnowski HW Coen DM Cullen BR MicroRNAs expressed by herpes simplex virus 1 during latent infection regulate viral mRNAs Nature 2008 454 780 783 18596690 10.1038/nature07103 PMC2666538

[B74] Perng G-C Jones C Ciacci-Zanella J Stone M Henderson G Yukht A Slanina SM Hofman FM Ghiasi H Nesburn AB Wechsler SL Virus-Induced Neuronal Apoptosis Blocked by the Herpes Simplex Virus Latency-Associated Transcript Science 2000 287 1500 1503 10.1126/science.287.5457.1500 10688801

[B75] Tang S Patel A Krause PR Novel Less-Abundant Viral MicroRNAs Encoded by Herpes Simplex Virus 2 Latency-Associated Transcript and Their Roles in Regulating ICP34.5 and ICP0 mRNAs J Virol 2009 83 1433 1442 10.1128/JVI.01723-08 19019961 PMC2620902

[B76] Margolis TP Imai Y Yang L Vallas V Krause PR Herpes Simplex Virus Type 2 (HSV-2) Establishes Latent Infection in a Different Population of Ganglionic Neurons than HSV-1: Role of Latency-Associated Transcripts J Virol 2007 81 1872 1878 10.1128/JVI.02110-06 17151134 PMC1797553

[B77] Kramer MF Coen DM Quantification of transcripts from the ICP4 and thymidine kinase genes in mouse ganglia latently infected with herpes simplex virus J Virol 1995 69 1389 1399 7853471 10.1128/jvi.69.3.1389-1399.1995 PMC188725

[B78] Feldman LT Ellison AR Voytek CC Yang L Krause P Margolis TP Spontaneous molecular reactivation of herpes simplex virus type 1 latency in mice Proc Natl Acad Sci 2002 99 978 983 10.1073/pnas.022301899 11773630 PMC117416

[B79] Liu T Khanna KM Chen X Fink DJ Hendricks RL Cd8+ T Cells Can Block Herpes Simplex Virus Type 1 (HSV-1) Reactivation from Latency in Sensory Neurons J Exp Med 2000 191 1459 1466 10.1084/jem.191.9.1459 10790421 PMC2213436

[B80] Khanna KM Bonneau RH Kinchington PR Hendricks RL Herpes simplex virus-specific memory CD8+ T cells are selectively activated and retained in latently infected sensory ganglia Immunity 2003 18 593 603 10.1016/S1074-7613(03)00112-2 12753737 PMC2871305

[B81] St. Leger AJ Peters B Sidney J Sette A Hendricks RL Defining the Herpes Simplex Virus-Specific CD8+ T Cell Repertoire in C57BL/6 Mice J Immunol 2011 186 3927 3933 10.4049/jimmunol.1003735 21357536 PMC3308013

[B82] Sheridan BS Cherpes TL Urban J Kalinski P Hendricks RL Reevaluating the CD8 T-Cell Response to Herpes Simplex Virus Type 1: Involvement of CD8 T Cells Reactive to Subdominant Epitopes J Virol 2009 83 2237 2245 10.1128/JVI.01699-08 19073721 PMC2643732

[B83] Ramachandran S Davoli KA Yee MB Hendricks RL Kinchington PR Delaying the Expression of Herpes Simplex Virus Type 1 Glycoprotein B (gB) to a True Late Gene Alters Neurovirulence and Inhibits the gB-CD8+ T-Cell Response in the Trigeminal Ganglion J Virol 2010 84 8811 8820 10.1128/JVI.00496-10 20573821 PMC2919033

[B84] Zerboni L Sobel RA Ramachandran V Rajamani J Ruyechan W Abendroth A Arvin A The expression of varicella-zoster virus immediate early regulatory protein IE63 in neurons of latently infected human sensory ganglia J Virol 2010 84 3421 3430 10.1128/JVI.02416-09 20106930 PMC2838126

[B85] Gowrishankar K Slobedman B Cunningham AL Miranda-Saksena M Boadle RA Abendroth A Productive Varicella-Zoster Virus Infection of Cultured Intact Human Ganglia J Virol 2007 81 6752 6756 10.1128/JVI.02793-06 17409155 PMC1900131

[B86] Markus A Grigoryan S Sloutskin A Yee MB Zhu H Yang IH Thakor NV Sarid R Kinchington PR Goldstein RS Varicella-Zoster Virus (VZV) Infection of Neurons Derived from Human Embryonic Stem Cells: Direct Demonstration of Axonal Infection, Transport of VZV, and Productive Neuronal Infection J Virol 2011 85 6220 6233 10.1128/JVI.02396-10 21525353 PMC3126485

[B87] Wang K Lau TY Morales M Mont EK Straus SE Laser-capture microdissection: refining estimates of the quantity and distribution of latent herpes simplex virus 1 and varicella-zoster virus DNA in human trigeminal Ganglia at the single-cell level J Virol 2005 79 14079 14087 10.1128/JVI.79.22.14079-14087.2005 16254342 PMC1280223

[B88] Cohrs RJ Barbour M Gilden DH Varicella-zoster virus (VZV) transcription during latency in human ganglia: detection of transcripts mapping to genes 21, 29, 62, and 63 in a cDNA library enriched for VZV RNA J Virol 1996 70 2789 2796 8627753 10.1128/jvi.70.5.2789-2796.1996 PMC190136

[B89] Lungu O Annunziato PW Gershon A Staugaitis SM Josefson D LaRussa P Silverstein SJ Reactivated and latent varicella-zoster virus in human dorsal root ganglia Proc Natl Acad Sci U S A 1995 92 10980 10984 10.1073/pnas.92.24.10980 7479921 PMC40554

[B90] Cohen JI Krogmann T Bontems S Sadzot-Delvaux C Pesnicak L Regions of the varicella-zoster virus open reading frame 63 latency-associated protein important for replication in vitro are also critical for efficient establishment of latency J Virol 2005 79 5069 5077 10.1128/JVI.79.8.5069-5077.2005 15795292 PMC1069579

[B91] Cohen JI Krogmann T Pesnicak L Ali MA Absence or overexpression of the Varicella-Zoster Virus (VZV) ORF29 latency-associated protein impairs late gene expression and reduces VZV latency in a rodent model J Virol 2007 81 1586 1591 10.1128/JVI.01220-06 17151102 PMC1797561

[B92] Cohen JI Krogmann T Ross JP Pesnicak L Prikhod’ko EA Varicella-zoster virus ORF4 latency-associated protein is important for establishment of latency J Virol 2005 79 6969 6975 10.1128/JVI.79.11.6969-6975.2005 15890936 PMC1112154

[B93] Cohrs RJ Barbour MB Mahalingam R Wellish M Gilden DH Varicella-zoster virus (VZV) transcription during latency in human ganglia: prevalence of VZV gene 21 transcripts in latently infected human ganglia J Virol 1995 69 2674 2678 7884921 10.1128/jvi.69.4.2674-2678.1995 PMC188953

[B94] Hoover SE Cohrs RJ Rangel ZG Gilden DH Munson P Cohen JI Downregulation of varicella-zoster virus (VZV) immediate-early ORF62 transcription by VZV ORF63 correlates with virus replication in vitro and with latency J Virol 2006 80 3459 3468 10.1128/JVI.80.7.3459-3468.2006 16537613 PMC1440367

[B95] Lungu O Panagiotidis CA Annunziato PW Gershon AA Silverstein SJ Aberrant intracellular localization of Varicella-Zoster virus regulatory proteins during latency Proc Natl Acad Sci U S A 1998 95 7080 7085 10.1073/pnas.95.12.7080 9618542 PMC22745

[B96] Mahalingam R Wellish M Cohrs R Debrus S Piette J Rentier B Gilden DH Expression of protein encoded by varicella-zoster virus open reading frame 63 in latently infected human ganglionic neurons Proc Natl Acad Sci U S A 1996 93 2122 2124 10.1073/pnas.93.5.2122 8700895 PMC39920

[B97] Sadzot-Delvaux C Arvin AM Rentier B Varicella-zoster virus IE63, a virion component expressed during latency and acute infection, elicits humoral and cellular immunity J Infect Dis 1998 178 Suppl 1 S43 S47 9852972 10.1086/514259

[B98] Xia D Srinivas S Sato H Pesnicak L Straus SE Cohen JI Varicella-zoster virus open reading frame 21, which is expressed during latency, is essential for virus replication but dispensable for establishment of latency J Virol 2003 77 1211 1218 10.1128/JVI.77.2.1211-1218.2003 12502838 PMC140846

[B99] Cohrs RJ Gilden DH Prevalence and abundance of latently transcribed varicella-zoster virus genes in human ganglia J Virol 2007 81 2950 2956 10.1128/JVI.02745-06 17192313 PMC1866015

[B100] Ambagala AP Bosma T Ali MA Poustovoitov M Chen JJ Gershon MD Adams PD Cohen JI Varicella-zoster virus immediate-early 63 protein interacts with human antisilencing function 1 protein and alters its ability to bind histones h3.1 and h3.3 J Virol 2009 83 200 209 10.1128/JVI.00645-08 18971269 PMC2612320

[B101] Di Valentin E Bontems S Habran L Jolois O Markine-Goriaynoff N Vanderplasschen A Sadzot-Delvaux C Piette J Varicella-zoster virus IE63 protein represses the basal transcription machinery by disorganizing the pre-initiation complex Biol Chem 2005 386 255 267 15843171 10.1515/BC.2005.031

[B102] Walters MS Kyratsous CA Wan S Silverstein S Nuclear import of the varicella-zoster virus latency-associated protein ORF63 in primary neurons requires expression of the lytic protein ORF61 and occurs in a proteasome-dependent manner J Virol 2008 82 8673 8686 10.1128/JVI.00685-08 18562514 PMC2519623

[B103] Stallings CL Duigou GJ Gershon AA Gershon MD Silverstein SJ The cellular localization pattern of Varicella-Zoster virus ORF29p is influenced by proteasome-mediated degradation J Virol 2006 80 1497 1512 10.1128/JVI.80.3.1497-1512.2006 16415026 PMC1346923

[B104] Stallings CL Silverstein SJ Posttranslational modification and cell type-specific degradation of varicella-zoster virus ORF29p J Virol 2006 80 10836 10846 10.1128/JVI.00966-06 16956951 PMC1641786

[B105] Farnsworth A Goldsmith K Johnson DC Herpes Simplex Virus Glycoproteins gD and gE/gI Serve Essential but Redundant Functions during Acquisition of the Virion Envelope in the Cytoplasm J Virol 2003 77 8481 8494 10.1128/JVI.77.15.8481-8494.2003 12857917 PMC165244

[B106] Johnson DC Spear PG Monensin inhibits the processing of herpes simplex virus glycoproteins, their transport to the cell surface, and the egress of virions from infected cells J Virol 1982 43 1102 1112 6292453 10.1128/jvi.43.3.1102-1112.1982 PMC256222

[B107] Cohrs RJ Mehta SK Schmid DS Gilden DH Pierson DL Asymptomatic reactivation and shed of infectious varicella zoster virus in astronauts J Med Virol 2008 80 1116 1122 10.1002/jmv.21173 18428120 PMC2938738

[B108] Gilden D Cohrs RJ Mahalingam R Nagel MA Neurological disease produced by varicella zoster virus reactivation without rash Curr Top Microbiol Immunol 2010 342 243 253 10.1007/82_2009_3 20186614 PMC3076592

[B109] Freeman ML Sheridan BS Bonneau RH Hendricks RL Psychological Stress Compromises CD8+ T Cell Control of Latent Herpes Simplex Virus Type 1 Infections J Immunol 2007 179 322 328 17579052 10.4049/jimmunol.179.1.322 PMC2367250

[B110] Cherpes TL Busch JL Sheridan BS Harvey SAK Hendricks RL Medroxyprogesterone Acetate Inhibits CD8+ T Cell Viral-Specific Effector Function and Induces Herpes Simplex Virus Type 1 Reactivation J Immunol 2008 181 969 975 18606648 10.4049/jimmunol.181.2.969 PMC2553693

[B111] Decman V Kinchington PR Harvey SAK Hendricks RL Gamma Interferon Can Block Herpes Simplex Virus Type 1 Reactivation from Latency, Even in the Presence of Late Gene Expression J Virol 2005 79 10339 10347 10.1128/JVI.79.16.10339-10347.2005 16051826 PMC1182646

[B112] Knickelbein JE Khanna KM Yee MB Baty CJ Kinchington PR Hendricks RL Noncytotoxic Lytic Granule-Mediated CD8+ T Cell Inhibition of HSV-1 Reactivation from Neuronal Latency Science 2008 322 268 271 10.1126/science.1164164 18845757 PMC2680315

[B113] Jiang X Chentoufi AA Hsiang C Carpenter D Osorio N BenMohamed L Fraser NW Jones C Wechsler SL The Herpes Simplex Virus Type 1 Latency-Associated Transcript Can Protect Neuron-Derived C1300 and Neuro2A Cells from Granzyme B-Induced Apoptosis and CD8 T-Cell Killing J Virol 2011 85 2325 2332 10.1128/JVI.01791-10 21177822 PMC3067767

[B114] Himmelein S St Leger A Knickelbein J Rowe A Freeman M Hendricks R Circulating herpes simplex type 1 (HSV-1)-specific CD8+ T cells do not access HSV-1 latently infected trigeminal ganglia Herpesviridae 2011 2 5 10.1186/2042-4280-2-5 21429183 PMC3070622

[B115] Chapman RS Cross KW Fleming DM The incidence of shingles and its implications for vaccination policy Vaccine 2003 21 2541 2547 10.1016/S0264-410X(03)00034-3 12744889

[B116] Camarena V Kobayashi M Kim JY Roehm P Perez R Gardner J Wilson AC Mohr I Chao MV Nature and Duration of Growth Factor Signaling through Receptor Tyrosine Kinases Regulates HSV-1 Latency in Neurons Cell Host Microbe 2010 8 320 330 10.1016/j.chom.2010.09.007 20951966 PMC2988476

[B117] Whitlow ZW Kristie TM Recruitment of the Transcriptional Coactivator HCF-1 to Viral Immediate-Early Promoters during Initiation of Reactivation from Latency of Herpes Simplex Virus Type 1 J Virol 2009 83 9591 9595 10.1128/JVI.01115-09 19570863 PMC2738264

[B118] Roxas M Herpes zoster and postherpetic neuralgia: diagnosis and therapeutic considerations Altern Med Rev 2006 11 102 113 16813460

[B119] Gowrishankar K Steain M Cunningham AL Rodriguez M Blumbergs P Slobedman B Abendroth A Characterization of the Host Immune Response in Human Ganglia after Herpes Zoster J Virol 2010 84 8861 8870 10.1128/JVI.01020-10 20573825 PMC2919016

[B120] Hasnie FS Breuer J Parker S Wallace V Blackbeard J Lever I Kinchington PR Dickenson AH Pheby T Rice AS Further characterization of a rat model of varicella zoster virus-associated pain: Relationship between mechanical hypersensitivity and anxiety-related behavior, and the influence of analgesic drugs Neuroscience 2007 144 1495 1508 10.1016/j.neuroscience.2006.11.029 17197105 PMC2394505

[B121] Garry EM Delaney A Anderson HA Sirinathsinghji EC Clapp RH Martin WJ Kinchington PR Krah DL Abbadie C Fleetwood-Walker SM Varicella zoster virus induces neuropathic changes in rat dorsal root ganglia and behavioral reflex sensitisation that is attenuated by gabapentin or sodium channel blocking drugs Pain 2005 118 97 111 10.1016/j.pain.2005.08.003 16213091

[B122] Gebhardt T Whitney PG Zaid A Mackay LK Brooks AG Heath WR Carbone FR Mueller SN Different patterns of peripheral migration by memory CD4+ and CD8+ T cells Nature 2011 477 216 219 10.1038/nature10339 21841802

[B123] Zerboni L Sobel RA Lai M Triglia R Steain M Abendroth A Arvin A Apparent expression of varicella-zoster virus proteins in latency resulting from reactivity of murine and rabbit antibodies with human blood group a determinants in sensory neurons J Virol 2012 86 1 578 583 10.1128/JVI.05950-11 22013055 PMC3255922

[B124] Ouwendijk WJ Choe A Nagel MA Gilden D Osterhaus AD Cohrs RJ Verjans GM Restricted varicella-zoster virus transcription in human trigeminal Ganglia obtained soon after death J Virol 2012 Sept 86 81 10203 10206 22740396 10.1128/JVI.01331-12 PMC3446590

